# Effects of crowberry (*Empetrum nigrum* subsp. *hermaphroditum*) supplementation on inflammatory and metabolic adverse effects in high-fat diet-induced experimental obesity

**DOI:** 10.29219/fnr.v70.14418

**Published:** 2026-10-01

**Authors:** Anni Alatalo, Mari Hämäläinen, Riitta Ryyti, Rainer Peltola, Katriina Vuolteenaho, Eeva Moilanen

**Affiliations:** 1 The Immunopharmacology Research Group, Faculty of Medicine and Health Technology, Tampere University and Tampere University Hospital, Wellbeing Services County of Pirkanmaa, Tampere, Finland; 2 Natural Resources Institute Finland, Bioeconomy and Environment, Rovaniemi, Finland

**Keywords:** obesity, low-grade systemic inflammation, hepatic inflammation, non-alcoholic fatty liver disease, crowberry

## Abstract

**Background:**

Obesity continues to be a major global health challenge, often accompanied by chronic low-grade inflammation and metabolic complications, including insulin resistance, impaired glucose metabolism, dyslipidemia and non-alcoholic fatty liver disease (NAFLD). Diets enriched with plant-derived polyphenols have shown promise in alleviating inflammation and promoting a metabolically healthier obesity phenotype. Crowberry, a polyphenol-rich wild berry native to the boreal forest zone, is particularly abundant in anthocyanins. Here, we investigated the effects of crowberry supplementation on low-grade inflammation and metabolic disturbances in a mouse model of diet-induced obesity.

**Design:**

Mice were fed for 12 weeks with low-fat or high-fat diet (10% or 46 % energy from fat, respectively), or with high-fat diet supplemented with crowberry powder. To ensure comparability, protein and micronutrient contents were standardized across all diets, and the two high-fat diets were matched for fat, carbohydrate, and fiber composition.

**Results:**

Crowberry supplementation markedly suppressed the high-fat diet-induced elevations in serum amyloid A (SAA) and alanine aminotransferase (ALT), markers of systemic and hepatic inflammation, respectively. Additionally, crowberry supplemented diet attenuated the expression of multiple pro-inflammatory genes both in the liver and visceral adipose tissue. Overall body weight and visceral fat gain were not altered by crowberry suggesting direct health-promoting effects by crowberry constituent.

**Conclusions:**

These findings demonstrate that crowberry supplementation can effectively counteract high-fat diet-induced systemic and hepatic inflammation in experimental obesity, supporting its use as a part of a healthy diet. Clinical studies are needed to assess the translation of these findings to humans and the role of crowberries in dietary strategies combating obesity and its comorbidities.

## Popular scientific summary

- Polyphenol-rich diet has been reported to attenuate low-grade inflammation and shift obesity towards a metabolically healthier phenotype.
- Crowberry is a polyphenol-rich, under-researched wild berry grown in boreal forest zone.
- In the present study, crowberry alleviated low-grade inflammation without affecting weight gain in diet-induced experimental obesity.
- High-fat diet-induced adverse changes in gene expression were prevented in the liver and adipose tissue.
- Crowberry supplementation may promote a healthier obesity phenotype as a part of balanced diet.

The prevalence of obesity and its comorbidities have increased significantly during the last decades. According to the WHO statistics, adult obesity has doubled, and childhood obesity has quadrupled since 1990. WHO estimates that 2.5 billion adults were overweight (body mass index [BMI] > 25 kg/m^2^) and 890 million of them were obese (BMI > 30 kg/m^2^) in 2022 (1). The growing prevalence of obesity is the result of changing lifestyle, which has led to an increase in obesogenic diets and a decrease in physical activity. Higher-than-optimal BMI is related to low-level systemic inflammation and metabolic dysregulation due to excessive adipose tissue. These inflammatory and metabolic changes may contribute to the development of obesity-related conditions such as insulin resistance, impaired glucose metabolism, hypertension, dyslipidemia, coronary heart disease and non-alcoholic fatty liver disease (NAFLD) (2, 3).

Adipose tissue functions as an endocrine and metabolic organ, secreting adipokines and cytokines and participating in glucocorticoid and steroid metabolism. Excessive lipid accumulation leads to metabolic, hypoxic and inflammatory changes in the adipose tissue, causing systemic low-grade inflammation. Systemic inflammation affects the regulation of nutrient metabolism in adipose tissue as well as in liver, pancreas, and muscle tissue, thus contributing to insulin resistance. In systemic low-grade inflammation, circulating concentrations of biomarkers such as serum amyloid A (SAA) rise. SAA further promotes the development of insulin resistance and increased SAA concentrations have also been associated with elevated cardiovascular disease risk (4, 5).

The treatment and prevention of obesity and its comorbidities are based on weight loss, physical activity, and healthy diet (2). Importantly, the risk of obesity-related diseases can also be reduced by alleviating systemic low-grade inflammation even without noticeable weight loss (4). It has been reported that a subset of individuals with obesity maintain insulin sensitivity, do not develop features of metabolic syndrome, and exhibit little or no adipose tissue inflammation. This is defined as a metabolically healthy obesity (MHO). Individuals with MHO are at a lower risk of developing type 2 diabetes (T2D) and hepatic inflammation than those with metabolically unhealthy obesity (MUHO). Therefore, interventions that promote a shift from MUHO phenotype toward MHO may help to reduce the adverse health consequences of obesity (4).

Diet has a major impact on the prevention and management of obesity and its comorbidities (2). Vegetables and berries rich in polyphenols, particularly anthocyanins and proanthocyanidins, are known to have antioxidant and anti-inflammatory properties (6). A diet abundant in plant-derived polyphenols has been shown to reduce low-grade inflammation and promote a metabolically healthier phenotype in obesity (7, 8). In previous studies, berries rich in polyphenolic compounds such as bilberries (*Vaccinium myrtillus* L.) and lingonberries (*Vaccinium vitis-idaea* L.) were found to support healthier inflammatory and metabolic state in obesity (9–13).

Crowberry (*Empetrum nigrum* subsp. *hermaphroditum*) is a high-yielding, underutilized wild berry that grows in the boreal forest zone. Beyond compositional analyses, relatively little is known about the biological effects of crowberry, particularly those of its tetraploid subspecies (*Empetrum nigrum* subsp. *hermaphroditum*), which grows farther north than the other subspecies. In previous studies, crowberry subsp. *hermaphroditum* has been reported to have antimicrobial and subsp. *japonicum* anticancer and anti-inflammatory properties *in vitro* and/or *in vivo* (14, 15). The composition of the crowberry has similarities with the more widely studied bilberry, which suggests that it may have similar biological effects than bilberry. Notably, crowberry, particularly its northern tetraploid subspecies (*Empetrum nigrum* subsp. *hermaphroditum*) contains significantly higher amounts of anthocyanins than bilberries. Anthocyanins are polyphenolic compounds that previous studies have suggested to largely contribute to the health benefits of berries (16–20).

In this study, we supplemented high-fat diet with crowberry powder and investigated its effects on obesity-associated inflammatory and metabolic adverse effects in an experimental mouse model. The results demonstrated that crowberry supplement mitigated obesity-related low-grade inflammation, particularly in the liver, without significantly affecting weight gain.

## Materials and methods

### Animals and study design

Eight-week-old male C57BL/6N mice were divided into three groups of 12 animals and maintained for 12 weeks on either high-fat (HF) or low-fat (LF) diet (46 and 10% energy from fat, respectively) or on HF diet supplemented with 20% (w/w) of air-dried crowberry powder. The 20% (w/w) dose of crowberry powder was selected based on widely used experimental protocols to allow comparison with previous studies (10, 12). Body weight and feed consumption of the mice were recorded weekly, and at the conclusion of the experiment, tissue and blood samples were harvested for later analysis. Throughout the study, the animals (two mice per cage) were housed under standard conditions in the preclinical research facilities of Tampere University (temperature 22 ± 1°C, 12 h light/dark cycle, 50–60% humidity). Mice had ad libitum access to food and water.

Feed pellets were customized to take into consideration the nutrient composition of crowberry powder as shown in Tables 1 and 2 (Research Diets, Inc., New Brunswick, NJ, USA). The protein, trace element, and vitamin contents of all feeds were in line with each other. In addition, the HF diets were matched in terms of carbohydrate, fat, and fiber content. Crowberry (*Empetrum nigrum* subsp. *hermaphroditum*) powder was made from Finnish crowberries. To produce 100 g of air-dried powder, approximately 900 g of fresh crowberries were used (Kiantama Oy, Suomussalmi, Finland).

**Table 1:** The macronutrient composition of the experimental diets

Nutrients	Low-fat diet (LF)		High-fat diet (HF)		Crowberry supplemented high-fat diet (HF + CRB)	
Calculated energy	kcal	kcal %	kcal	kcal %	kcal	kcal %
Protein	178 kcal	18	179 kcal	18	179 kcal	18
Carbohydrate	720 kcal	72	361 kcal	36	361 kcal	36
Fat	102 kcal	10	460 kcal	46	460 kcal	46
Total energy	1,000 kcal	100	1,000 kcal	100	1,000 kcal	100

Calculated energy per gram of diet (kcal/g)	3.6 kcal/g		4.4 kcal/g		4.4 kcal/g	

**Table 2:** Ingredients (g/1,000 g) of the experimental diets

Ingredients (g/1,000 g)	Low-fat diet (LF)	High-fat diet (HF)	Crowberry supplemented high-fat diet (HF + CRB) (CRB 200 g/1,000 g)
Crowberry powder (g)	0	0	200
Casein (protein) (g)	182	222	210 (+12 from CRB), total 222
L-Cystine (g)	2.7	3.3	3.3
Corn starch (g)	389	81	51 (+30 from CRB), total 81
Maltodextrin 10 (g)	91	111	110 (+1 from CRB), total 111
Glucose (g)	79	96	76 (+20 from CRB), total 96
Fructose (g)	79	96	75 (+21 from CRB), total 96
Sucrose (g)	0.6	0.7	0.1 (+0.6 from CRB), total 0.7
Cellulose (insoluble fiber) (g)	81	98	0 (+98 from CRB), total 98
Inulin (soluble fiber) (g)	3.6	4.3	4.3
Soybean oil (g)	23	28	8 (+20 from CRB), total 28
Lard (g)	18	200	200
Mineral mix S10026 (g)	9	11	11
Vitamin mix V10001 (g)	9	11	11
DiCalcium phosphate (g)	12	14	14
Potassium citrate (g)	15	18	18
Calcium carbonate (g)	5	6	6
Choline bitartrate (g)	1.8	2.2	2.2

The study (license no. ESAVI/984/04.10.07/2018) was authorized by the National Animal Experimental Board, and the experiments were conducted in accordance with EU legal regulations (Directive 2010/63/EU).

### Intraperitoneal glucose tolerance test

An intraperitoneal glucose tolerance test (IPGTT) was performed 1 week before the end of the study (at the week 11). In the morning, the mice underwent a 6-h fasting period, after which fasting blood glucose was measured from the peripheral tail vein using a Contour Next One glucometer (Oy Diabet Ab, Lemu, Finland). Thereafter, the mice were dosed intraperitoneally with sterile glucose dissolved in phosphate-buffered saline (2 g/kg body weight; Sigma-Aldrich, St. Louis, MO, USA), and blood glucose levels were determined at 30, 60, 90, 120, and 180 min following the glucose injection.

### Blood samples and analyses

Upon completion of the study, the mice were fasted for 6 h (morning fast), and blood glucose levels were determined from the peripheral tail vein. Subsequently, blood was collected via cardiac puncture under isoflurane anesthesia, and tissue samples (liver and epididymal fat) were collected. Blood samples were incubated for 45 min at r.t. before serum was separated by centrifugation and stored at –80°C until analyzed.

Serum alanine aminotransferase (ALT) activity and triglyceride and cholesterol concentrations were determined by fluorometric assays (Abcam, Cambridge, UK). Serum concentrations of insulin (Mercodia Ltd., Uppsala, Sweden), as well as those of SAA, adipsin, adiponectin, leptin and resistin (R&D Systems Europe Ltd., Abingdon, UK), were measured using enzyme immunoassays.

### RNA extraction and qRT-PCR

Liver tissue samples (25–30 mg) were homogenized with Precellys homogenizer (Bertin Technologies, Montigny-le-Bretonneux, France), and RNA was extracted with GeneElute RNA extraction kit (Sigma-Aldrich, St. Louis, MO, USA). Epididymal fat tissue samples (80–120 mg) were lysed in QIAzol reagent (Qiagen Inc., Hilden, Germany), and RNeasy Lipid Tissue Kit was used in RNA extraction (Qiagen). Both RNA extraction procedures included on-column DNase digestion.

RNA was reverse transcribed into complementary DNA (cDNA) using the Maxima First Strand cDNA Synthesis Kit (Thermo Fisher Scientific, Waltham, MA, USA). Polymerase chain reaction (PCR) was carried out as described previously (10). In-house primers and probes were obtained from Metabion (Martinsried, Germany) for the housekeeping gene glyceraldehyde 3-phosphate dehydrogenase (*Gapdh*) (10, 21). TaqMan Gene Expression assays (Thermo Fisher Scientific) were used to analyze the genes of interest, as listed in Table 3.

**Table 3:** TaqMan gene expression assays used in the study

Gene	Abbr.	Assay ID
Adiponectin	Adipoq	Mm00456425_m1
Adipsin	Cfd/Adn	Mm01143935_g1
Chemokine (C-X-C motif) ligand 14	Cxcl14	Mm00444699_m1
Cytochrome P450, family 3, subfamily a, polypeptide 11	Cyp3a11	Mm00731567_m1
Insulin like growth factor binding protein 2	Igfbp2	Mm00492632_m1
Leptin	Lep	Mm00434759_m1
Leptin receptor	Lepr	Mm00440181_m1
Mannose receptor C type 2	Mrc2	Mm00485184_m1
Metallothionein 1	Mt1	Mm00496660_g1
Monocyte chemoattractant protein 1	Mcp1	Mm00441242_m1
Resistin	Retn	Mm00445641_m1
S100 calcium-binding protein A8	S100a8	Mm00496696_g1
S100 calcium-binding protein A10	S100a10	Mm00501458_g1
Serum amyloid A1	Saa1	Mm00656927_g1
Serum amyloid A2	Saa2	Mm04208126_mH
Serum amyloid A3	Saa3	Mm00441203_m1

### Statistics

The data are expressed as mean ± standard error of the mean (SEM). Tukey’s IQR method (22) was used to identify possible outliers. When calculating the IPGTT results, the area under the curve (AUC) was determined using the trapezoidal method in GraphPad Prism 9 software (Graph Pad Software, San Diego, USA). The baseline was defined by the fasting glucose level of the LF group, and AUC was calculated in the range of 0–180 min. HOMA-IR (homeostatic model assessment for insulin resistance) values were calculated as previously described (19). The calculations followed a previously described model (23), where fasting plasma insulin (mU/L; conversion factor from (24)) was multiplied by fasting plasma glucose (mmol/L) and the result was divided by the constant 22.5.)

Statistical analyses were performed using one-way and two-way analyses of variance (ANOVA), followed by Bonferroni’s post hoc test for multiple comparisons. A *P*-value below 0.05 was considered statistically significant. Significance levels were indicated as follows: *P < 0.05; **P < 0.01; ***P < 0.001. GraphPad Prism 9 was used for both statistical analysis and graph generation.

## Results

### Weight gain and feed consumption

Mice were fed for 12 weeks with low-fat diet (LF diet, 10% of energy from fat), high-fat diet (HF diet, 46% of energy from fat), or with crowberry powder supplemented HF diet (HF + CRB diet). HF diet significantly increased body weight and visceral fat gain (*P* < 0.001, compared to LF diet), but they were not affected by crowberry supplementation (Fig. 1A–C).

**Fig. 1:**
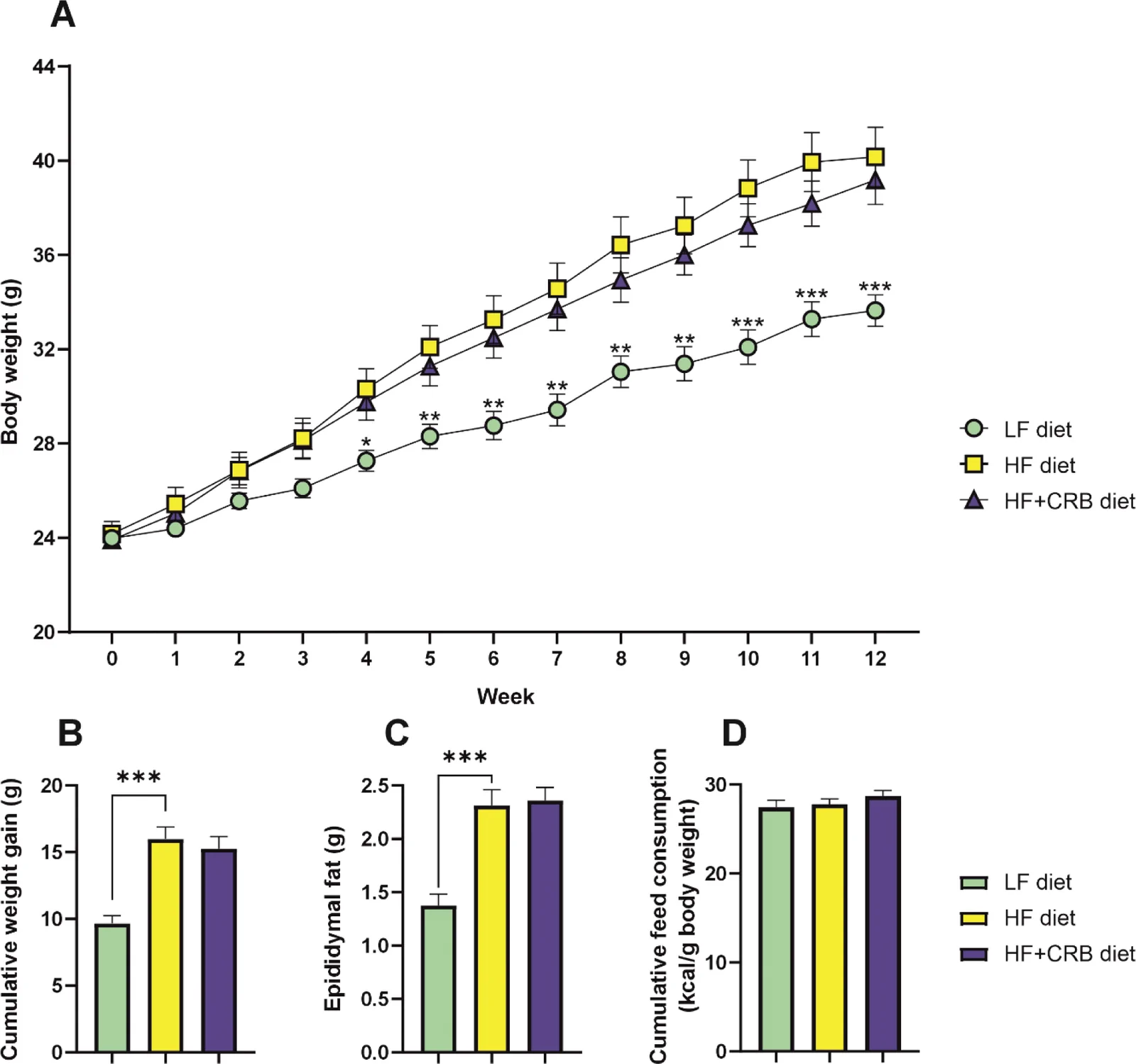
Weight gain (A) and cumulative weight gain (B) during the study, epididymal adipose tissue mass at study endpoint (C) and cumulative feed consumption (D). The mice received a low-fat diet (LF, 10% energy from fat, green), high-fat diet (HF, 46% energy from fat, yellow) or high-fat diet supplemented with crowberry (HF + CRB, blue). Weight and feed consumption were measured weekly. Significant differences in mean weight compared to the high-fat group are marked with asterisks *P < 0.05, **P < 0.01, and ***P < 0.001. Values are presented as mean ± standard error of the mean (SEM), with *n* = 12 mice per group. Statistical analyses were performed using one-way analyses of variance (ANOVA) (B–D) and repeated measures two-way ANOVA (A), both with Bonferroni post hoc correction.

Feed consumption (kcal / mouse body weight) was measured weekly (Fig. 1D). The high-fat diet groups (HF diet and HF + CRB diet) did not differ in feed consumption during the study in weekly measurements. No differences in cumulative feed consumption were found between the groups (Fig. 1D).

### SAA and ALT

SAA is a widely used biomarker of low-grade systemic inflammation in experimental obesity. SAA levels increased significantly in the HF diet group compared to the LF diet group (*P* < 0.01), being 717 ± 32 ng/mL in the HF diet group and 496 ± 62 ng/mL in the LF diet group. The SAA levels in the HF + CRB diet group (312 ± 31 ng/mL) were significantly lower than those in the HF diet group (*P* < 0.001), indicating that crowberry supplementation prevented the HF diet-induced increase in SAA levels and systemic inflammation (Fig. 2A).

**Fig. 2:**
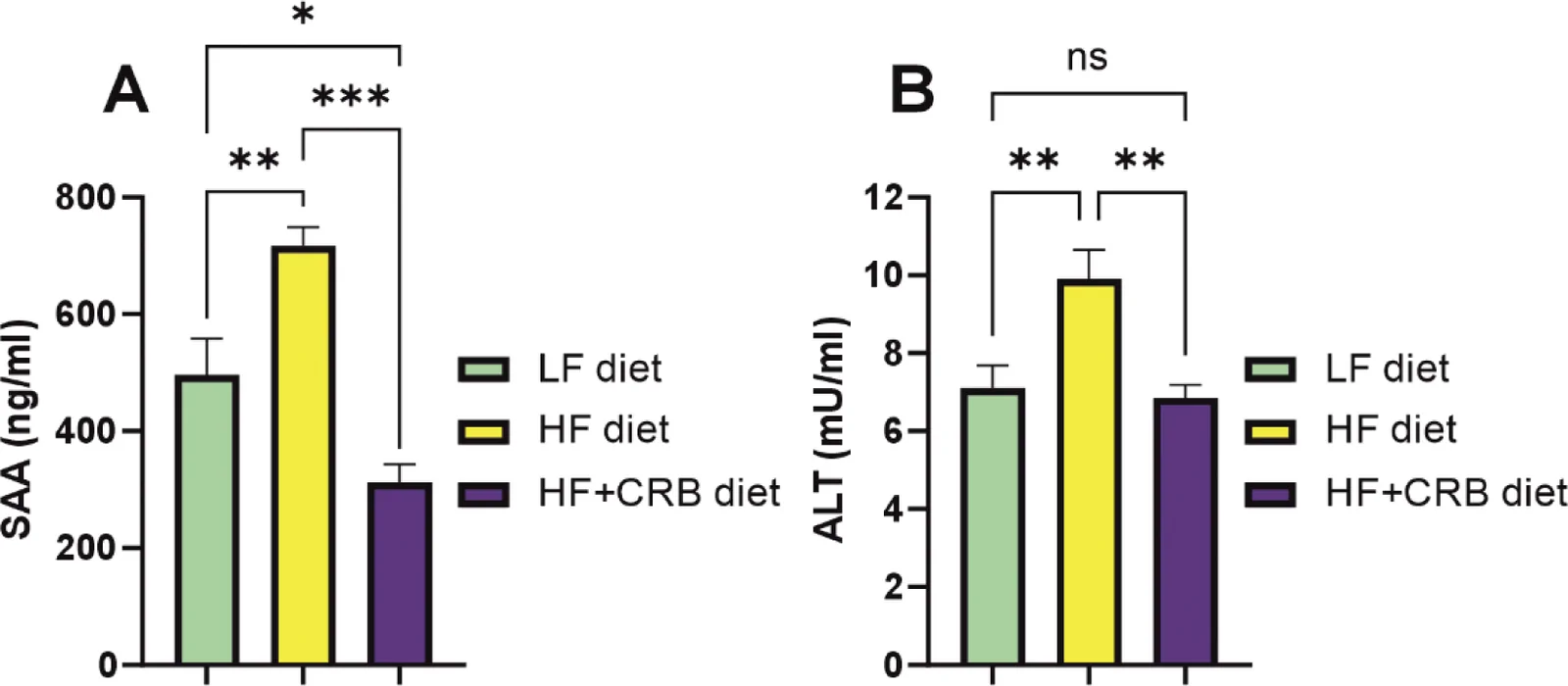
The circulating levels of serum amyloid A (SAA) (A) and serum alanine aminotransferase (ALT) activity (B) in mice, measured using an enzyme-linked immunosorbent assay (A) and a fluorometric assay (B) from serum at study endpoint. The mice were assigned to one of three dietary groups: a low-fat diet (LF, 10% energy from fat, green), high-fat diet (HF, 46% energy from fat, yellow) or high-fat diet supplemented with crowberry (HF + CRB, blue). Statistically significant differences in mean weight compared to the high-fat group are indicated by asterisks: *P < 0.05, **P < 0.01, ***P < 0.001, ns = not significant. Values are presented as mean + standard error of the mean (SEM), with *n* = 11–12 mice per group. Statistical analyses were conducted using one-way analysis of variance (ANOVA), followed by Bonferroni post hoc correction.

Serum concentration of ALT is a commonly used biomarker of hepatic inflammation and injury. High-fat diet induced a meaningful increase in circulating ALT levels compared to the LF diet (*P* < 0.01), being 9.90 ± 0.75 mU/mL in the HF diet group and 7.11 ± 0.58 mU/mL in the LF diet group. Crowberry supplementation completely prevented the HF diet-induced increase in ALT levels (*P* < 0.01), being 6.80 ± 0.33 mU/mL; no statistically significant difference in ALT levels were seen between the LF and HF + CRB diet groups (Fig. 2B). These results indicate that crowberry supplementation has a preventive effect on the HF diet-induced increase in circulating ALT enzyme levels and may reduce liver inflammation and injury associated with HF diet.

### Gene expression in the liver

High-fat diet is known to induce changes in gene expression in the liver (21). As illustrated in Fig. 3, the HF diet increased the expression of the inflammatory genes *Saa1, Saa2, Mcp-1, Cxcl14* and *S100a10* in a statistically significant manner compared to the LF diet (*P* < 0.05 for all the above). Crowberry supplementation to the HF diet prevented the increase in the expression of *Saa1*, *Saa2, Mcp-1* and *Cxcl14* in a statistically significant manner, and their expressions in the HF + CRB diet group did not differ from that in the LF diet group. The HF + CRB diet showed a trend toward moderating *S100a10* expression (*P* = 0.086), but the difference between HF and HF + CRB groups did not reach statistical significance.

**Fig. 3:**
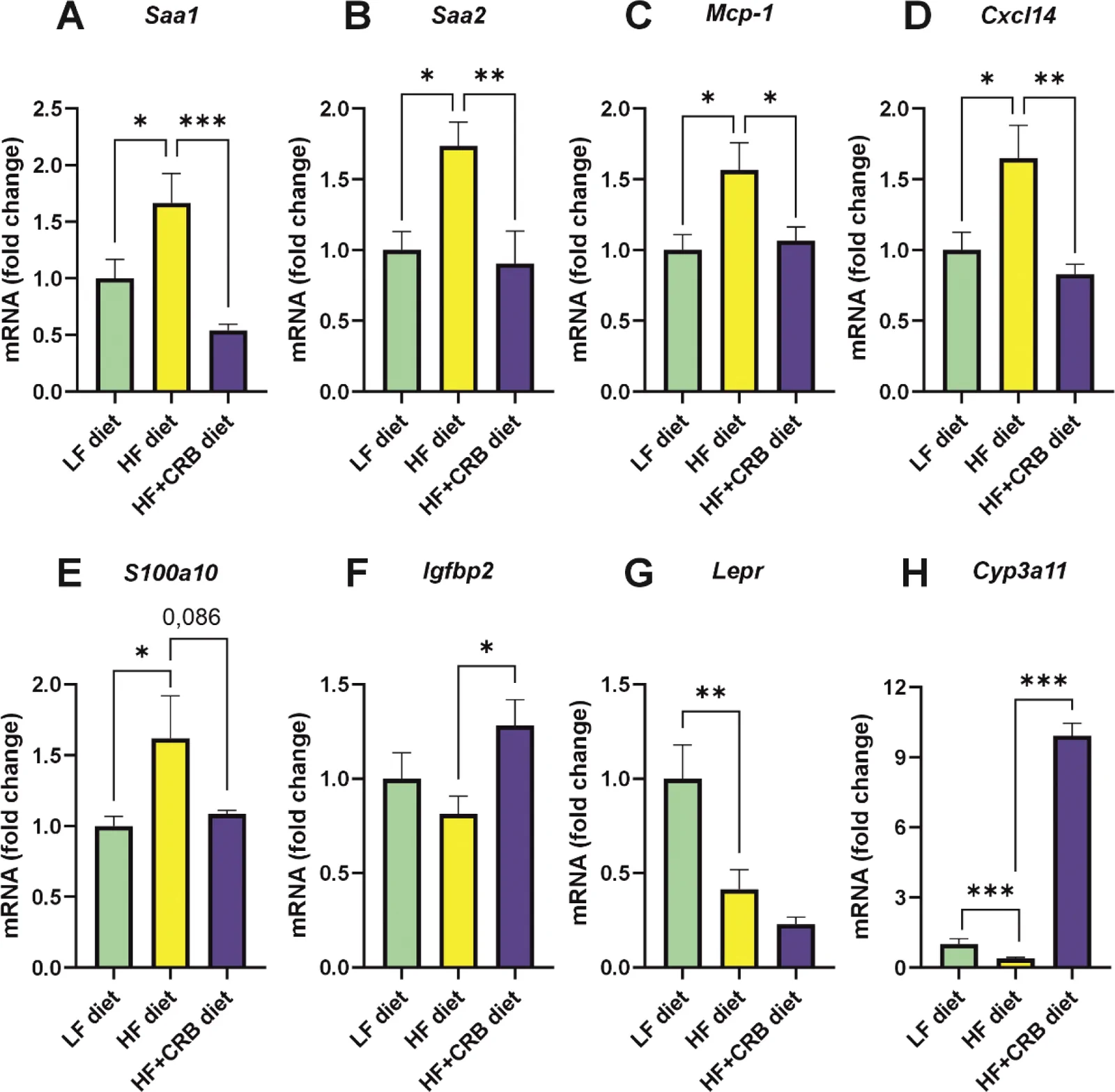
Gene expression in the liver analyzed by quantitative reverse transcription polymerase chain reaction (RT-PCR). The mice were assigned to one of three dietary groups: a low-fat diet (LF, 10% energy from fat, green), high-fat diet (HF, 46% energy from fat, yellow) or high-fat diet supplemented with crowberry (HF + CRB, blue). The expression data from RT-PCR were normalized to the housekeeping gene (Gapdh). Expression levels are presented as fold changes, with the LF group set as the reference (set to 1). Statistically significant differences are indicated by asterisks: *P < 0.05, **P < 0.01, ***P < 0.001. Values are presented as mean + standard error of the mean (SEM), with *n* = 11–12 mice per group. Statistical analyses were conducted using one-way analysis of variance (ANOVA), followed by Bonferroni post hoc correction. *Gapdh* = Glyceraldehyde 3-phosphate dehydrogenase, *Saa1* = Serum amyloid A1, *Saa2* = Serum amyloid A2, *Mcp-1* = Monocyte chemoattractant protein 1, *Cxcl14* = Chemokine (C-X-C motif) ligand 14, *S100a10* = S100 calcium-binding protein A10, *Igfbp2* = Insulin-like growth factor binding protein 2, *Lepr* = Leptin receptor, *Cyp3a11* = Cytochrome P450, family 3, subfamily a, polypeptide 11.

In addition, we measured the hepatic expression of insulin sensitivity-protecting *Igfbp2* (insulin growth factor binding protein 2), leptin receptor (*Lepr*) and *Cyp3a11* (cytochrome P450 (CYPs), family 3, subfamily a, polypeptide 11), which is involved e.g. in lipid metabolism. High-fat diet had a minor if any effect on *Igfbp2* expression, but its expression was significantly increased by crowberry supplementation (*P* < 0.05). High-fat diet decreased the expression of *Lepr* compared to the LF diet (*P* < 0.001) and that was not affected by crowberry supplementation. *Cyp3a11* expression decreased in the HF diet group compared to LF diet group (*P* < 0.001) and a significant increase was seen in the HF + CRB group (*P* < 0.001).

### Gene expression in the visceral adipose tissue and adipokines

High-fat diet is known to cause adverse metabolic and inflammatory changes in the adipose tissue. As shown in Fig. 4, the HF diet increased the expression of *Saa3* (*P* < 0.001), *Mcp-1* (*P* < 0.001), *Cxcl14* (*P* < 0.01), *S100a8* (*P* < 0.001) and *Mt1* (*P* < 0.001) in a statistically significant manner compared to the LF diet. In addition, the HF diet showed an increasing trend in *Mrc2* gene expression (*P* = 0.0632), but the difference to the LF diet did not reach statistical significance. Crowberry supplementation to the HF diet prevented the increase in *Saa3, Mcp-1* and *Mt1* expression compared to the control HF diet (*P* < 0.05 for all the above), and *Saa3* expression did not differ from the LF group. In addition, the HF + CRB diet group showed a decreasing trend in *Cxcl14* (*P* = 0.0812) expression, but the difference to the HF diet did not reach statistical significance.

**Fig. 4:**
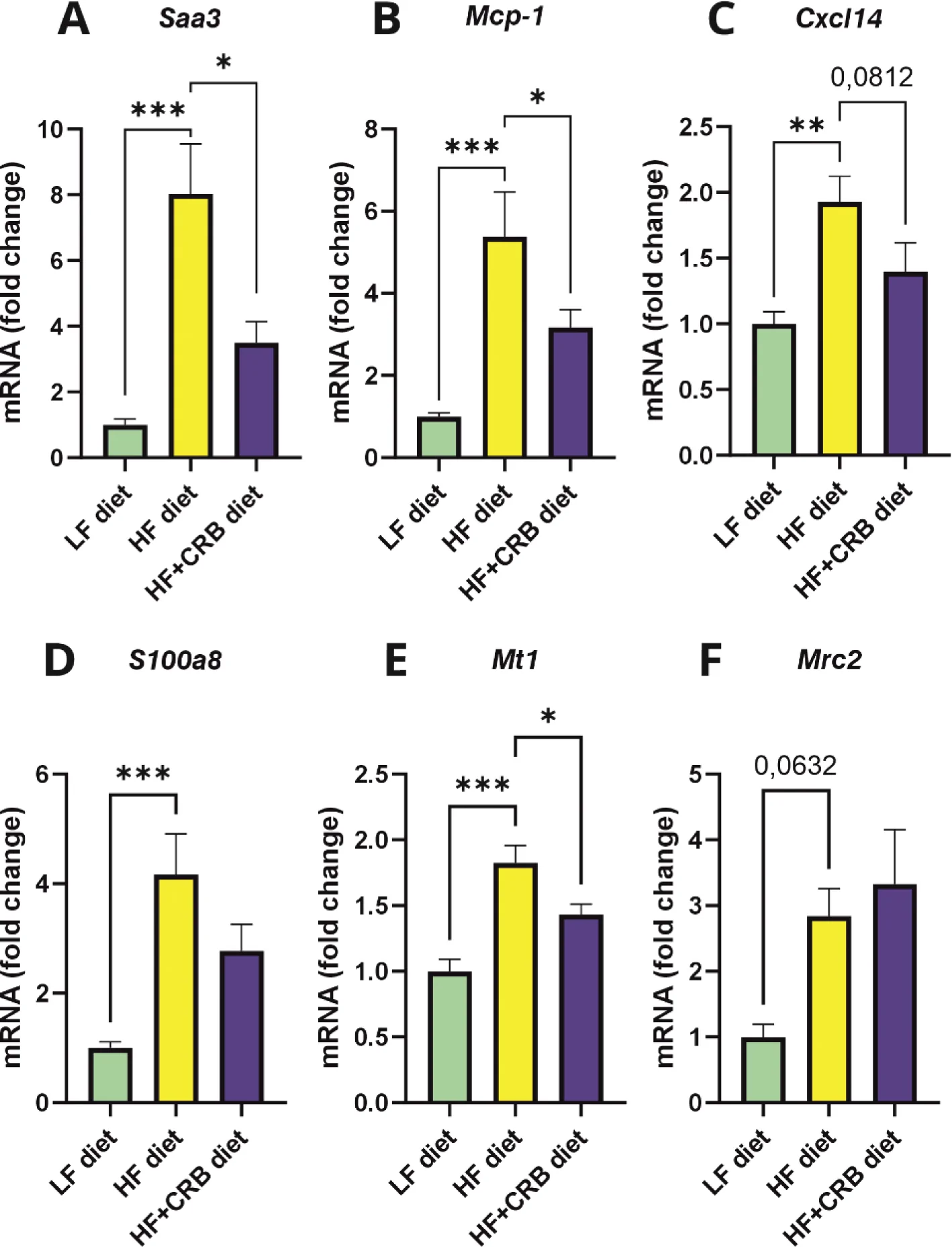
Gene expression in epididymal adipose tissue analyzed by quantitative reverse transcription polymerase chain reaction (RT-PCR). The mice were assigned to one of three dietary groups: a low-fat diet (LF, 10% energy from fat, green), high-fat diet (HF, 46% energy from fat, yellow) or high-fat diet supplemented with crowberry (HF + CRB, blue). The expression data from RT–PCR were normalized to the housekeeping gene (Gapdh). Expression levels are presented as fold changes, with the LF group set as the reference (set to 1). Statistically significant differences are indicated by asterisks: *P < 0.05, **P < 0.01, ***P < 0.001. Values are presented as mean + standard error of the mean (SEM), with *n* = 10–12 mice per group. Statistical analyses were conducted using one-way analysis of variance (ANOVA), followed by Bonferroni post hoc correction. *Gapdh* = Glyceraldehyde 3-phosphate dehydrogenase, *Saa3* = Serum amyloid A3, *Mcp-1* = Monocyte chemoattractant protein 1, *Cxcl14* = Chemokine (C-X-C motif) ligand 14, *S100a8* = S100 calcium-binding protein A8, *Mt1* = Metallothionein 1, *Mrc2* = Mannose receptor C type 2.

Adipokines are, by definition, factors secreted by adipose tissue to regulate appetite and energy metabolism. In addition, they are known to be produced by hepatic and some other tissues and to be involved in inflammatory responses. As shown in Table 4, leptin levels in serum increased (*P* < 0.001), and adiponectin levels decreased (*P* < 0.05) in a statistically significant manner in the HF diet group compared to the LF diet group.

**Table 4:** Serum adipokine levels of mice at the end of the study

Adipokine	Low-fat diet (LF)	High-fat diet (HF)	Crowberry supplemented high-fat diet (HF + CRB)
Leptin (ng/mL)	8.21 ± 1.10	34.62 ± 2.13***	26.57 ± 3.50
Adiponectin (µg/mL)	7.70 ± 0.66	5.89 ± 0.43*	6.80 ± 0.51
Adipsin (mg/mL)	9.60 ± 0.56	7.36 ± 0.24***	8.86 ± 0.32^#^
Resistin (ng/mL)	16.53 ± 0.62	18.88 ± 1.07	18.10 ± 0.74

Serum adipokine consentrations were measured by ELISA. The results are presented as mean ± SEM; *n* = 11–12 mice per group. Statistical analysis was performed using one-way ANOVA with Bonferroni’s post-test.

Mean values in the HF group that differ significantly from the LF group are indicated by *P < 0.05, and ***P < 0.001; mean value in the HF + CRB group that differs significantly from the HF group is indicated by ^#^*P* < 0.05.

SEM: standard error of the mean; ANOVA: analyses of variance; ELISA: enzyme-linked immunosorbent assay.

The crowberry-supplemented HF diet group had a higher adiponectin but a lower leptin level than the HF group (Table 4), but the differences were not statistically significant. High-fat diet decreased serum adipsin levels compared to the LF diet (*P* = 0.001) and that was significantly moderated by crowberry supplementation (*P* < 0.05). Serum resistin levels did not differ between the groups.

### Lipids and glucose metabolism

High-fat diet enhanced cholesterol levels in a statistically significant manner (*P* < 0.01), being 2.62 ± 0.07 mmol/L in the HF group and 2.03 ± 0.16 mmol/L in the LF group (Fig. 5A). Crowberry supplementation had no effect on cholesterol levels. There were no statistically significant differences in triglyceride levels between the groups (Fig. 5B).

**Fig. 5:**
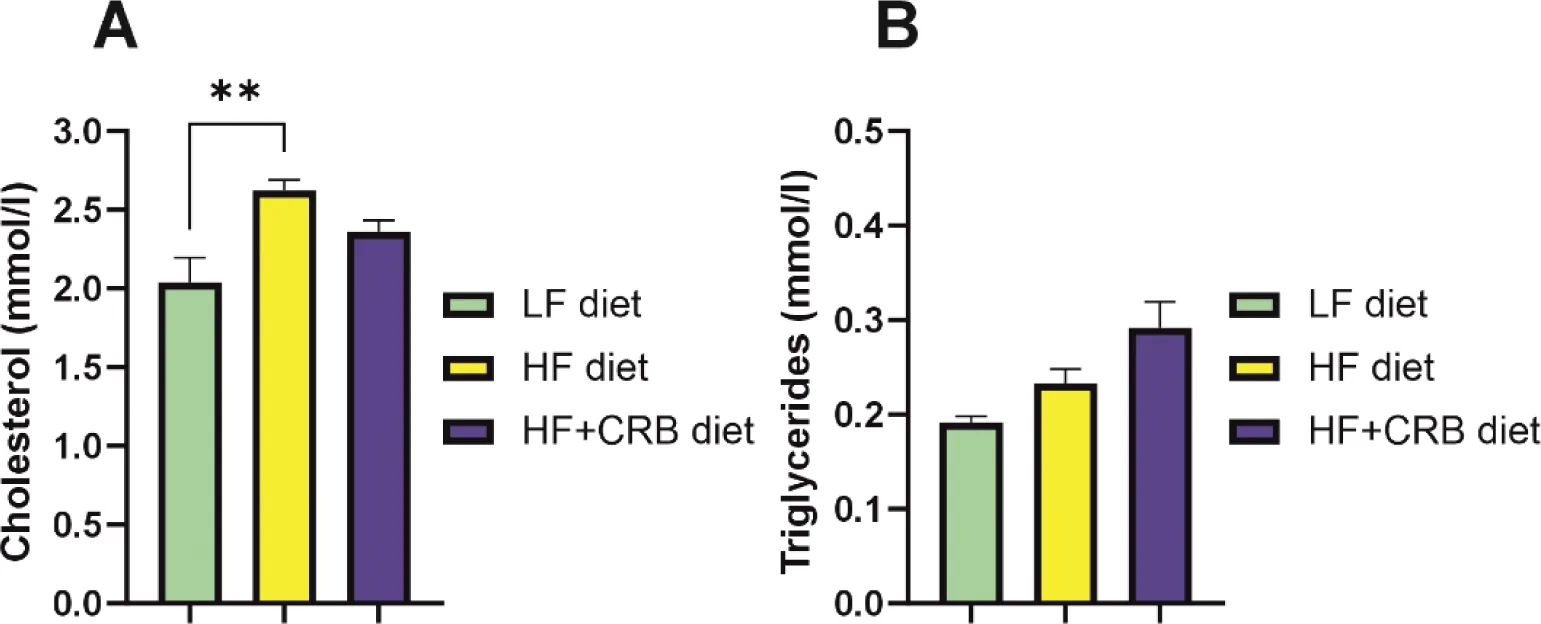
Cholesterol (A) and triglyceride (B) levels in mice measured using a fluorometric assay from serum at the study endpoint. The mice were assigned to one of three dietary groups: a low-fat diet (LF, 10% energy from fat, green), high-fat diet (HF, 46% energy from fat, yellow) or high-fat diet supplemented with crowberry (HF + CRB, blue). Statistically significant differences in mean weight compared to the high-fat group are indicated by asterisks: **P < 0.01. Values are presented as mean + standard error of the mean (SEM), with *n* = 11–12 mice per group. Statistical analyses were conducted using one-way analysis of variance (ANOVA), followed by Bonferroni post hoc correction.

At week 11 of the 12-week study, an IPGTT was carried out as a measure of glucose metabolism. In the IPGTT, there were no differences between the groups in the fasting glucose levels at the onset of the test. At the remaining time points, both the HF group and the HF + CRB group had statistically significantly higher blood glucose levels than the LF diet group (Fig. 6A). In the HF diet groups, blood glucose levels spiked higher after glucose administration than in the LF diet group and returned more slowly to baseline than in the LF group. There was no statistically significant difference between the HF group and the HF + CRB group at any time point. The AUC method gave similar results, with the HF group having a significantly higher AUC compared to the LF diet group (*P* < 0.001). Crowberry supplementation to the HF diet had no effect on the AUC.

**Fig. 6:**
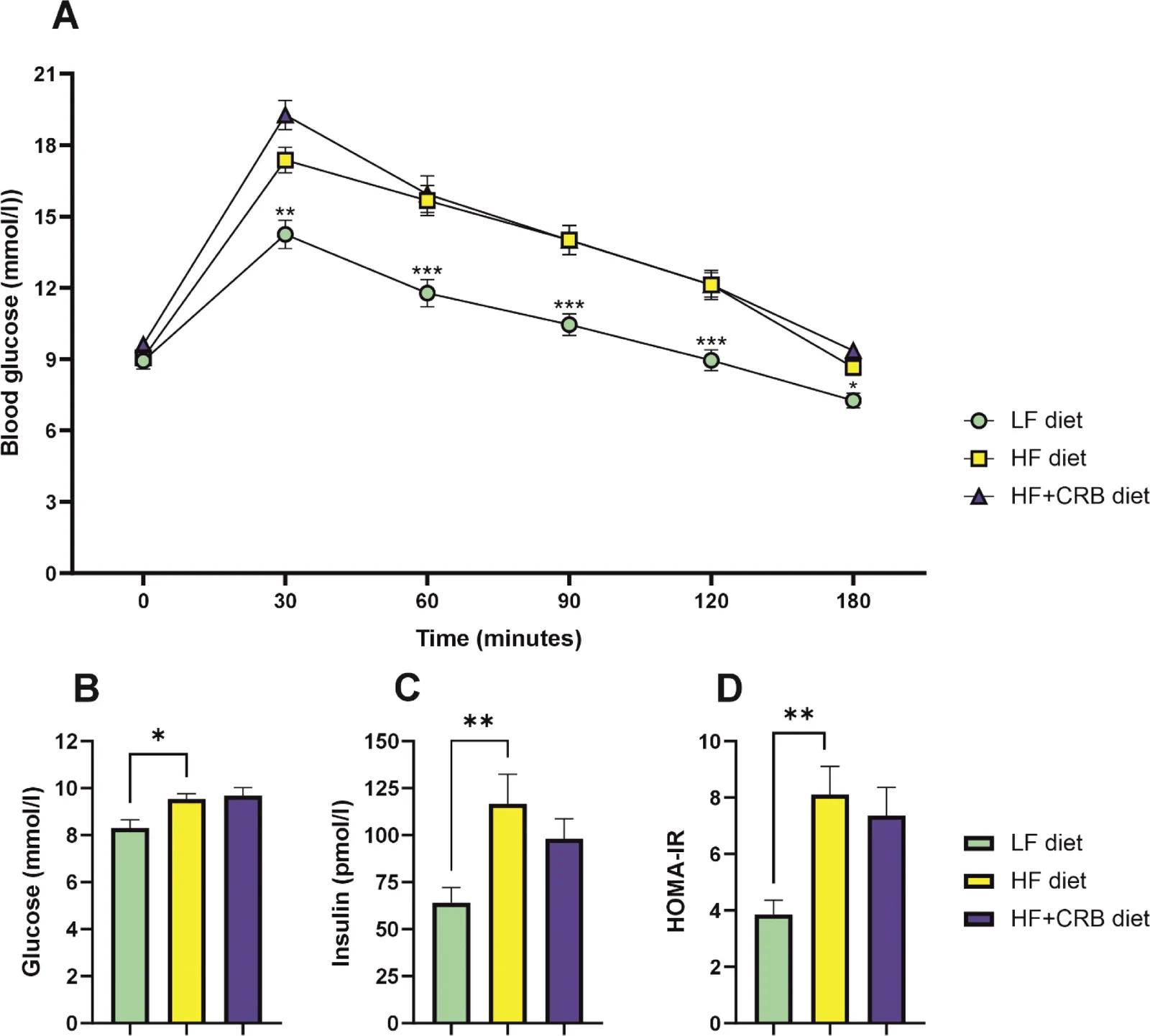
Intraperitoneal glucose tolerance test (IPGTT) (A) was performed at week 11 of the 12-week study, and fasting blood glucose levels (B), insulin levels (C), and the homeostatic model assessment for insulin resistance index (HOMA-IR index) (D) were measured at the study endpoint. The mice were assigned to one of three dietary groups: a low-fat diet (LF, 10% energy from fat, green), high-fat diet (HF, 46% energy from fat, yellow) or high-fat diet supplemented with crowberry (HF + CRB, blue). In the IPGTT mice received a glucose bolus, which was 2 mg/g. Blood glucose levels were measured at the indicated time points from the tip of the tail (peripheral tail vein) using a Contour Next One meter (Oy Diabet Ab, Lemu, Finland) and insulin using an enzyme-linked immunosorbent assay from serum at study endpoint. Statistically significant differences in mean weight compared to the high-fat group are indicated by asterisks: *P < 0.05, **P < 0.01, ***P < 0.001. Values are presented as mean + standard error of the mean (SEM), with *n* = 10–12 mice per group. Statistical analyses were conducted using one-way analysis of variance (ANOVA), followed by Bonferroni post hoc correction.

Fasting blood glucose was also measured at week 12, when the mice were terminated. At the end of the study the HF diet had higher fasting blood glucose levels (*P* < 0.05) compared to the LF diet, being 9.54 ± 0.23 mmol/L in the HF diet group and 8.31 ± 0.35 mmol/L in the LF diet group (Fig. 6B). Crowberry supplementation to the HF diet had no effect on the fasting blood glucose levels.

The HF diet significantly increased insulin levels (*P* < 0.01) compared to the LF diet, being 116.70 ± 15.82 pmol/L in the HF diet group and 64.03 ± 8.13 pmol/L in the LF diet group. Mean insulin levels in the HF + CRB diet group were lower (98.15 ± 10.48 pmol/L) than those in the HF diet group, but the difference was not statistically significant (Fig. 6C). Correspondingly, the HOMA-IR index was significantly higher (*P* < 0.01) in the HF diet group (8.11 ± 0.99) than in the LF diet group (3.85 ± 0.51). The crowberry-supplemented HF diet group had somewhat lower HOMA-IR levels (7.36 ± 1.01) than the HF diet group, but the difference was not statistically significant (Fig. 6D).

## Discussion

The effects of air-dried crowberry (*Empetrum nigrum* subsp. *hermaphroditum*) powder on HF diet-induced inflammatory and metabolic changes were investigated in a mouse model of experimental obesity. Crowberry supplementation prevented the HF diet-induced increases in the circulating levels of the inflammatory biomarkers SAA and ALT, as well as the expression of inflammation-related genes in epididymal adipose tissue and liver. These findings suggest that crowberry supplementation attenuated systemic and hepatic inflammation associated with HF diet-induced obesity. There was no difference in weight gain or energy intake between the HF diet groups. Therefore, the observed differences are most likely due to crowberry supplementation in the diet and not, for example, to less severe obesity. Thus, the crowberry supplementation in the diet seems to be able to shift obesity toward a healthier phenotype, which is associated with reduced risk of comorbidities in obesity (4).

Obesity is associated with systemic low-grade inflammation, which contributes to the metabolic comorbidities associated with obesity. SAA was used in this study as a marker of systemic inflammation. In experimental mouse models, SAA is widely used instead of C-reactive protein (CRP), which is the most commonly used inflammatory biomarker in humans; because CRP is not a major acute-phase protein in mice and its synthesis is low (10, 13, 25). Four subtypes of SAA (SAA1-4) have been identified, of which SAA1-3 are increased in inflammation while SAA4 is constitutively expressed. SAA1 and SAA2 are mainly produced in liver and SAA3 in adipose tissue (4, 26). In this study, circulating levels of SAA and its expression in liver (*Saa1, Saa2*) and epididymal adipose tissue (*Saa3*) increased significantly during the HF diet feeding, whereas crowberry supplementation totally prevented these changes. These results suggest that crowberry supplementation prevents systemic inflammation induced by HF diet.

Obesity is associated with fat accumulation and inflammation in the liver, which may develop into NAFLD or further into more severe liver disease NASH (non-alcoholic steatohepatitis) (27). Serum/plasma ALT is a widely used biomarker of liver inflammation and injury. In this study, ALT levels were increased in mice on HF diet and crowberry supplementation fully prevented the change. Insulin resistance and excessive intake of fats and energy may lead to a dysfunction in the lipid-energy metabolism in the liver (28, 29). Part of the accumulated excessive fat is metabolized into toxic metabolites, leading to cellular stress and hepatocyte injury. The injured liver cells and activated Kupffer cells secrete proinflammatory cytokines and chemokines that accelerate inflammation and contribute to the development of fibrosis (28). Interestingly, a previous study showed, that four out of eight investigated berries, namely lingonberry, blackcurrant, bilberry and crowberry, decreased triglyceride and/or cholesterol accumulation in the liver in a mouse model of diet-induced obesity (12).

In this study, we found that crowberry supplementation attenuated HF diet-induced hepatic upregulation of several inflammatory genes. Changes in the mRNA expression of *Saa1, Saa2, Mcp-1* and *Cxcl14* were completely prevented by crowberry supplementation in mouse liver. A beneficial effect was also seen in the expression of *S100a10* and *Igfbp2*. These results indicate that crowberry supplementation to the HF diet can partially prevent the development of liver inflammation. In particular, the prevention of increased expression of the chemokines *Cxcl14* and *Mcp-1* likely reduces the recruitment of inflammatory cells to the liver, thereby alleviating the progression of the inflammatory process. Accordingly, Heyman-Lindén et al. have provided histological evidence demonstrating an increased accumulation of macrophages into the livers of HF diet-fed mice, which was attenuated in mice receiving lingonberries (13). Furthermore, the positive effect of crowberry supplementation on *Igfbp2* gene expression observed in this study, may be relevant, as the *Igfbp2* gene has been shown to play a protective role on insulin sensitivity (30).

Crowberry-containing diet was found to increase the expression of *Cyp3a11* in mouse liver. CYP3A11 is part of the CYPs enzyme family, which is involved in the metabolism of lipids and fatty acids. CYP enzymes also contribute to the synthesis and degradation of fat-soluble vitamins and hormones and participate in the detoxification of endogenous and exogenous compounds (31, 32). It has been shown that *Cyp3a11* expression is reduced in liver inflammation (33, 34). Accordingly, we found that HF diet decreased *Cyp3a11* expression while the HF + CRB diet had an increasing effect. Of note, mouse CYP3A11 has functional similarities to CYP3A4, an important drug-metabolizing enzyme in humans (33). Further studies are needed to understand the metabolic consequences (if any) of altered *Cyp3a11* expression induced by HF diet and/or crowberry supplementation.

Visceral adipose tissue has high metabolic activity, and its excessive accumulation is connected to impaired glucose metabolism and persistent low-level inflammation (4, 5). Epididymal adipose tissue has been generally used in mouse models of obesity as a tissue equivalent to visceral fat (10, 13, 35, 36). In this study, crowberry diet modulated gene expression not only in the liver but also in the epididymal adipose tissue. Crowberry supplementation completely prevented HF diet-induced increase in *Saa3* expression and significantly attenuated that in *Mcp-1* and *Mt1* expression. All these factors are involved in inflammation. The role of SAA was discussed above. Monocyte chemoattractant protein-1 (MCP-1) stimulates monocyte and macrophage infiltration and disrupts glucose metabolism in adipose tissue (37). MT1 is involved in oxidative stress, which promotes inflammation in adipose tissue (38). In addition, crowberry tended to prevent the increasing effect of HF diet on the expression of *Cxcl14* and *S100a8*. In general, the effect of crowberry supplementation on adipose tissue gene expression seems to be stronger than that of another anthocyanidin-rich wild berry, bilberry (10). As in many previous studies gene expression in adipose tissue and the liver was evaluated at the mRNA level, providing insight into the transcriptional effects of crowberry supplementation (10, 11, 36, 39). Further studies are needed to confirm the extent to which these changes are translated to the protein level.

Adipokines are bioactive factors secreted by adipose tissue and known to have inflammatory and regulatory effects (3, 37). In this study, leptin, adiponectin and adipsin concentrations were affected by HF diet. Crowberry significantly prevented the changes in adipsin levels, while a trend toward attenuation was observed for leptin and adiponectin levels. Interestingly, adipsin has been found to have a beta-cell protective effect and a moderating effect on hyperglycemia in a mouse model of T2D (40).

In this study, HF diet increased serum levels of cholesterol as expected and that was not affected by crowberry supplementation. Crowberry seeds contain fatty acids that could be expected to influence cholesterol levels (41). However, no such effect was observed, which may be explained by the balanced fatty acid composition across the high-fat diets. It would therefore be interesting to examine the effect of crowberry seed oil on cholesterol levels. Triglyceride levels often rise in humans as a result of a HF diet, while in mice triglyceride levels are generally not affected by HF diet or may even decrease (42, 43). In this study, neither HF diet nor crowberry supplementation had any statistically significant effect on triglyceride levels.

In this study, crowberry supplementation to the HF diet had no effect on glucose metabolism except for an increase in the hepatic expression of the insulin sensitivity protective gene *Igfbp2*. High-fat diet increased insulin levels and impaired glucose tolerance in the IPGTT, but the effect was rather moderate compared to previous studies (10, 11, 19), and no effect of crowberry was found on these parameters.

Crowberry is rich in phenolic compounds, especially anthocyanins and their aglycosides, which are called as anthocyanidins (17, 18, 44). Anthocyanins are color pigments formed in plants during the growing season, and they have been shown to have anti-inflammatory and antioxidant effects *in vivo* and *in vitro* (6). The anthocyanin profile of crowberry (*Empetrum nigrum* subsp. *hermaphroditum*) has similarities to that of the better studied wild berry bilberry (*Vaccinium myrtillus* L.) (18). Both berries have been reported to contain 15 anthocyanin glycoside groups, the main ones being cyanidin, delphinidin, petunidin, peonidin and malvidin (45). However, crowberries have a higher total anthocyanin content and, accordingly, stronger antioxidant activity than bilberries and many other well-studied berries such as blueberries, cranberries, blackberries, blackcurrants, redcurrants, mulberries, raspberries, and strawberries (18). Crowberry also contains flavanols such as quercetin and kaempferol and flavan-3-ols including epicatechin. In addition, crowberries harbor proanthocyanidins, although at significantly lower levels than lingonberries (46, 47). In a murine model of obesity, both bilberry and lingonberry supplementations have been shown to have beneficial effects on glucose and lipid metabolism, systemic inflammation and liver tissue inflammation, with lingonberry also shown to limit body weight and visceral fat gain (10, 11, 19, 21). The differences in the observed effects between berries in obesity models are most likely due to differences in their polyphenol contents.

Anthocyanins have been shown to have anti-inflammatory effects through several cellular mechanisms (48). They have been reported to inhibit TLR4 expression (49) as well as the activation of the NF-*κ*B (50) and MAP-kinase signaling pathways (51), thereby reducing the production of proinflammatory cytokines, eicosanoids, reactive oxygen species (ROS) and nitric oxide (NO) (48). In addition, anthocyanins cyanidin, delphinidin and malvidin have been shown to attenuate oxidative stress (52), fibrosis-related responses (53), hepatic lipid accumulation (54), and insulin resistance (55, 56), as well as to support intestinal barrier function and gut microbial balance (57, 58). These mechanisms may well explain, at least partly, the anti-inflammatory effects of crowberry seen in this study.

Relatively little research on crowberry exists beyond compositional analyses, and the studies do not always report which subspecies have been used. *In vitro* studies have shown that crowberry extract inhibits 15-lipoxygenase and xanthine oxidase, enzymes involved in ROS production, and stimulates glucose uptake in cultured human liver cells (59). In addition, crowberry juice and its derived compounds have been shown to inhibit bacterial adherence (14, 60–62). Crowberry extract has also demonstrated wound-healing properties, anti-angiogenic activity *in vitro* and *in vivo*, and inhibitory effects on cancer cell proliferation and migration via Akt and DEK signaling in a xenograft model (15, 63, 64). Previous study by Heyman et al. screened eight berry species, including crowberry, for their effects on body weight and metabolic abnormalities in diet-induced experimental obesity. In line with our findings, crowberry supplementation had no effect on weight gain or plasma glucose, insulin or lipid levels, but it reduced cholesterol accumulation in the liver (12).

This study extends previous findings by demonstrating that crowberry, more specifically its northern tetraploid subspecies *Empetrum nigrum* subsp. *hermaphroditum*, can counteract HF diet-induced systemic and hepatic inflammation in experimental obesity, but further studies are needed to evaluate the translatability of these findings to human health. Notably, this study suggests that crowberry supplementation may promote a healthier obesity phenotype through beneficially modulating inflammatory pathways. These findings provide a rationale for further investigating the potential role of crowberry, a berry abundant in boreal forests across northern regions, as part of a healthy diet.

## Conclusions

In conclusion, these findings show that crowberry supplementation attenuated the development of systemic and hepatic inflammation in HF diet-induced experimental obesity without affecting body weight or visceral fat gain. The findings suggest that crowberry supplementation may promote a healthier obesity phenotype as a part of balanced diet. Clinical studies are needed to assess the translation of these findings to humans and the role of crowberries in dietary strategies combating obesity and its comorbidities.
